# Genetic analysis of Japanese patients with small bowel adenocarcinoma using next-generation sequencing

**DOI:** 10.1186/s12885-022-09824-6

**Published:** 2022-07-02

**Authors:** Atsushi Tatsuguchi, Takeshi Yamada, Koji Ueda, Hiroyasu Furuki, Aitoshi Hoshimoto, Takayoshi Nishimoto, Jun Omori, Naohiko Akimoto, Katya Gudis, Shu Tanaka, Shunji Fujimori, Akira Shimizu, Katsuhiko Iwakiri

**Affiliations:** 1 Department of Gastroenterology, Nippon Medical School, 1-1-5 Sendagi, Bunkyo-ku, Tokyo, 113-8603 Japan; 2 Department of Analytic Human Pathology, Nippon Medical School, 1-1-5 Sendagi, Bunkyo-ku, Tokyo, 113-8602 Japan; 3 Department of Gastrointestinal and Hepato-Biliary-Pancreatic Surgery, Nippon Medical School, 1-1-5 Sendagi, Bunkyo-ku, Tokyo, 113-8603 Japan

**Keywords:** Genomic alterations, Japanese, MMR status, Mucin phenotype, Small bowel adenocarcinoma

## Abstract

**Background:**

Small bowel adenocarcinomas (SBAs) are rare and there is little comprehensive data on SBA genomic alterations for Asian patients. This study aimed to profile genomic alterations of SBA in Japanese patients using targeted next-generation sequencing (NGS).

**Methods:**

We examined 22 surgical resections from patients with primary SBA. SBA genomic alterations were analyzed by NGS. Mismatch repair (MMR) status was determined by immunohistochemical analysis. Mucin phenotypes were classified as gastric (G), intestinal (I), gastrointestinal (GI), and null (N) types on MUC2, MUC5AC, MUC6, and CD10 immunostaining.

**Results:**

The most common genomic alterations found in SBA tumors were *TP53* (*n* = 16), followed by *KRAS* (*n* = 6), *APC* (*n* = 5), *PIK3CA* (*n* = 4), *CTNNB1* (*n* = 3), *KIT* (*n* = 2)*, BRAF* (*n* = 2)*, CDKN2A* (*n* = 2)*,* and *PTEN* (*n* = 2). Deficient MMR tumors were observed in 6 out of 22 patients. Tumor mucin phenotypes included 2 in G-type, 12 in I-type, 3 in GI-type, and 5 in N-type. *APC* and *CTNNB1* mutations were not found in G-type and GI-type tumors. *KRAS* mutations were found in all tumor types except for G-type tumors. *TP53* mutations were found in all tumor types. Although no single gene mutation was associated with overall survival (OS), we found that *KRAS* mutations were associated with significant worse OS in patients with proficient MMR tumors.

**Conclusions:**

SBA genomic alterations in Japanese patients do not differ significantly from those reports in Western countries. Tumor localization, mucin phenotype, and MMR status all appear to impact SBA gene mutations.

**Supplementary Information:**

The online version contains supplementary material available at 10.1186/s12885-022-09824-6.

## Background

Small bowel cancers are rare, accounting for less than 5% of gastrointestinal cancers, but their incidence is increasing [1, 2]. Small bowel adenocarcinoma (SBA) accounts for one-third of small bowel cancers, and thus scant data exist on its molecular and clinicopathological features due to its rarity [1]. Furthermore, it is not clear whether cancers that arise in the duodenum, jejunum and ileum share common molecular pathological features [3–5].

Small bowel cancers are more likely to be diagnosed at a later stage since patients rarely notice symptoms in its early stage; furthermore, the small intestine is difficult to examine except for duodenum [6]. Despite the relatively high rate of SBA being found as unresectable advanced cancer of the small intestine, no specific chemotherapy regimen has been developed and instead treatment akin to that of colorectal cancer has been performed palliatively [7]. This is due in great part to the rarity of SBA and the lack of sufficient data on its molecular biological and clinicopathological features. More cases need to be evaluated to address these issues through comprehensive analyses of genomic SBA alterations, and new studies are beginning to come in reporting data on SBA gene mutations [8–12]. Schrock et al. performed a large-scale analysis comparing the genetic profiles of SBA, colorectal cancer (CRC), and gastric cancer (GC), and reported that SBA has a unique genetic profile that can be differentiated from GC and CRC [10]. A recent study examined genomic SBA profiles using a nationwide database from France and concluded there was no significant correlation between mutation rates and primary tumor localization, nor were genomic alterations associated with overall survival [12]. To date, studies have found certain mutated genes that commonly arise in SBA, including *TP53*, *KRAS*, *APC*, and *SMAD4*. However, there is a lack of consensus regarding individual gene mutation rates among studies. The relationship between individual gene mutations and mismatch repair (MMR) status and patients’ prognosis also remains controversial [11, 13, 14]. Moreover, there is a lack of comprehensive data on SBA genomic alterations reported from Asia, apart from one study on the genomic profile of Chinese SBA cohorts [15]. The purpose of this study is to investigate gene mutations in Japanese SBA patients by next-generation sequencing (NGS) and elucidate their molecular biological characteristics.

## Patients and methods

### Patients and tissue samples

We obtained 22 surgically resected small intestinal adenocarcinoma tissue samples from archives of the Department of Pathology at Nippon Medical School Hospital for genetic and immunohistochemical analysis [16]. Patients were eligible if they had a histologically confirmed diagnosis of SBA and both tumor and matched normal tissue samples were obtained. We excluded tumors of the ampullary region since they might have originated in the pancreas or the biliary tract. All subjects gave informed consent, and the project was approved by the Ethics Committee of Nippon Medical School. TNM staging criteria was defined according to the International Union Against Cancer TNM classifications.

### Immunohistochemical analysis

Immunohistochemical analysis was performed as described previously [16]. Specimens were fixed in 10% formalin, embedded in paraffin wax, and immersed in 0.5% H_2_O_2_–methanol for 10 min to block endogenous peroxidase activity. Sections were then microwaved in 0.01 mol/l citrate phosphate buffer (pH 6.0) or EDTA (pH9.0) for antigen retrieval and incubated with 10% normal horse or goat serum for 10 min at 37 °C to block nonspecific immunoglobulin (IgG) binding. Thereafter, sections were incubated for 18 h at 4 °C with anti-MLH1 (dilution 1:10; Biosciences, G168-15), anti-MSH2 (dilution 1:50; DAKO), anti-MSH6 (dilution 1:100; Leica), or anti-PMS2 (ready to use; Leica, EP51). They were then treated with their respective biotinylated antibodies; namely, anti-mouse IgG, or anti-rabbit IgG (dilution 1:200; Vector) for 30 min at 25 °C, followed by treatment with avidin–biotin peroxidase complex for 30 min at 25 °C. The reaction products were developed by immersing sections in 3,3 ‘-diaminobenzidine tetrahydrochloride solution containing 0.03% H_2_O_2_. The mucin phenotypes were classified into gastric (G), intestinal (I), gastrointestinal (GI), and null (N) types based on MUC2 (dilution 1:50; Dako, CCP58), MUC5AC (dilution 1:50, Dako, CLH2), MUC6 (ready to use; Leica, CLH5) and CD10 (dilution 1:100, Leica, 56C6) staining. Cases showing their immunoreactivites in over 10% of cancer cells were considered positive. Both MUC 2 and CD10 are markers of intestinal type tumors, whereas MUC5AC and MUC6 are both markers of gastric type tumors. Tissue samples showing both gastric and intestinal phenotypes were classified as GI type tumors, while those showing neither gastric nor intestinal phenotype expression were classified as N type tumors.

Tumors were considered negative when there was a complete absence of nuclear staining of neoplastic cells in the presence of an internal positive control assessed in a whole slide. Tumors with a negative staining of one of the MMR proteins were considered as deficient MMR (dMMR) and all others as proficient MMR (pMMR). In addition, immunostaining for HER2 (ready to use, Roche/Ventana, 4B5), p53 (dilution 1:50, Dako, DO-7), β-catenin (dilution 1:250, abcam, ab32572) was performed to compare the results of NGS.

### Next-generation sequencing (NGS)

DNA extraction, NGS, and mutation calling were performed as described previously [17]. DNA was purified from FFPE tumor tissue samples using a QIAamp DNA Mini kit (Qiagen, Limburg, the Netherlands) according to the manufacturer's recommendations. Total DNA concentration was measured using a NanoDrop™ ND-1000 Spectrophotometer (Thermo Fisher Scientific, Waltham, MA, USA). Multiplex polymerase chain reaction (PCR) and ligating adapters were performed using an Ion Chef™ system and an Ion AmpliSeq™ Kit for Chef DL8 (Thermo Fisher Scientific). Approximately 10 ng of DNA per sample was amplified using multiplex PCR with the Ion AmpliSeq™ Cancer Hotspot Panel v2 (Thermo Fisher Scientific), which is designed to amplify 207 amplicons covering the 2849 Catalogue of Somatic Mutations in Cancer (COSMIC; http://cancer.sanger.ac.uk/cosmic) mutations in hotspot regions for the 50 most commonly reported oncogenes and tumor suppressor genes. Pooled and barcoded libraries were clonally amplified on Ion Sphere™ particles using emulsion PCR with the Ion Chef™ system and Ion PGM™ HI-Q™ View Chef Kit (Thermo Fisher Scientific) according to the manufacturer's instructions. The subsequently enriched template-positive Ion Sphere™ particles were loaded onto an Ion 318™ chip and sequenced on an Ion Torrent™ PGM™ System (Thermo Fisher Scientific). Data from sequencing runs on the Ion Torrent™ PGM™ System were automatically transferred to the Torrent Server hosting the Torrent Suite™ Software v5.2.2 (Thermo Fisher Scientific). The Torrent Suite Software uses the Torrent Browser, which includes the Torrent Mapping Alignment Program and Torrent Variant Caller for alignment and variant detection. The variant calling was done with CHP2 Panel Somatic PGM using low stringency settings. To exclude miscalls, the results of raw variant calls of the Torrent Suite Software were manually checked using a visualization tool, the Integrative Genome Viewer. Germline mutations were excluded with reference to the Human Genetic Variation Database (http://www.genome.med.kyoto-u.ac.jp/SnpDB) [18]. Somatic mutations, which are considered to have no pathological significance or strongly related blood disease judged by reference to FATHMM predictions [19] and ClinVar (https://www.ncbi.nlm.nih.gov/clinvar/) [20], were also excluded. Only pathogenic or likely pathogenic variants according to CinVar were included. All the detected variants were confirmed by droplet digital PCR using QX200 Droplet Digital PCR system (Bio-Rad Laboratories, Hercules, California, USA).

### Statistical analysis

Genetic and Immunohistochemical results were compared with clinicopathological factors including age, gender, location, node metastasis (pN), depth of invasion (pT) and stage, using the chi-square test or Fisher’s exact test as appropriate. The association among each protein immunostaining was also assessed by the chi-square test or Fisher’s exact test as appropriate. The distribution of cancer-specific survival was estimated by Kaplan–Meier method, and the log-rank test was used to test for significant differences in cancer-specific overall survival. A *P* value of < 0.05 was considered significant.

## Results

### Clinicopathological characteristics of patients and tumors

Patient characteristics are presented in Table 1. Patients included 16 men and 6 women ranging in age from 32 to 84 years (average age, 62.0 years; median, 63 years). At the time of analysis, 10 patients had died. The median follow-up time for the whole series was 63 months (mean, 62.6 months; range, 17 to 115 months). Only one patient was classified as Lynch syndrome based on revised Bethesda guidelines. No other patients presented with predisposing conditions. Tumors were located in the duodenum in 3 cases, the jejunum in 16 cases and the ileum in 3 cases. All 3 tumors located in the duodenum were non-ampullary. Seven patients had distant metastases, six of them in the peritoneum and one in the lung. Multiple organ metastases were seen in 2 patients, with spleen/peritoneal and uterus/peritoneal metastases. Tumor mucin phenotypes included G-type (*n* = 2), I-type (*n* = 12), GI-type (*n* = 3) and N-type (*n* = 5).

**Table 1:** Clinicopathological and histological characteristics of patients with SBA

No	Sex	Age	Site	Histology	pT	pN	M	P	Stage	MMR	mucin phenotype
1	M	73	D	WD	1a	0	0	0	1	pMMR	intestinal
2	M	84	D	Muc	3	0	0	0	2A	pMMR	intestinal
3	M	59	J	WD	3	0	0	0	2A	dMMR	intestinal
4	M	32	J	WD	3	0	0	0	2A	dMMR	intestinal
5	M	61	J	WD	3	0	0	0	2A	dMMR	intestinal
6	F	74	J	PD	3	0	0	0	2A	pMMR	gastric
7	M	55	J	PD	3	0	0	0	2A	pMMR	gastrointestinal
8	M	82	J	WD	3	0	0	0	2A	pMMR	null
9	F	35	I	WD	3	0	0	0	2A	dMMR	intestinal
10	M	67	J	MD	3	0	0	0	2B	pMMR	null
11	M	74	D	MD	4	1	0	0	3A	pMMR	null
12	F	59	J	WD	3	1	0	0	3A	pMMR	null
13	M	83	J	PD	3	1	0	0	3A	dMMR	gastrointestinal
14	F	69	J	MD	3	1	0	0	3A	pMMR	intestinal
15	M	69	J	Muc	3	2	0	0	3B	pMMR	intestinal
16	M	39	J	MD	3	2	1	1	4	pMMR	null
17	M	44	J	WD	3	1	1	1	4	pMMR	gastric
18	M	52	J	PD	3	2	1	3	4	pMMR	intestinal
19	M	65	J	MD	3	2	2	1	4	pMMR	intestinal
20	F	73	J	MD	3	2	1	0	4	dMMR	gastrointestinal
21	F	61	I	PD	3	2	1	1	4	pMMR	intestinal
22	M	53	I	WD	4	2	1	1	4	pMMR	intestinal

*P* Peritoneum, *D* Duodenum, *J* Jejunum, *I* Ileum, *WD* Well differentiated, *MD* Moderately differentiated, *PD* Poorly differentiated, *Muc* Mucinous adenocarcinoma, *dMMR* DNA mismatch repair-deficient, *pMMR* DNA mismatch repair-proficient

### DNA mismatch repair status of tumors

MMR status was determined by means of immunohistochemical analysis. A negative staining was observed for both MLH1 and PMS2 in 4 tumors, and for MSH2 and MSH6 in 2 tumors. As a result, dMMR tumors were observed in 6 out of 22 patients. Comparisons of clinicopathological characteristics of patients according to MMR status are shown in additional file 1. dMMR tumors were associated with a younger age, non-mucinous, non-pT4 and a lower metastatic stage, although not statistically significant.

### The results of NGS

**Fig. 1:**
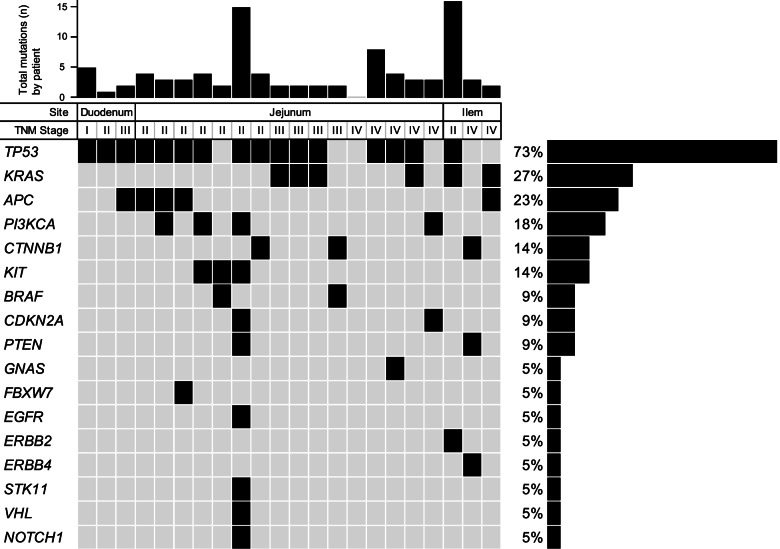
Mutational landscape of Japanese patients with small bowel adenocarcinoma. The somatic mutational profiles of all cases. The upper panel shows the numbers of total mutations in each tumor. The central plot shows the key clinical parameters, below which the recurrent mutated genes for each case are exhibited. The number of the recurrent mutated genes observed per tumor was 0 in 1, 1 in 3, 2 in 12 and > 2 in 6 patients. The median number of significant mutations for each patient was two (range; 0–6). The figure included 25 genes identified in SBA patients (*n* = 22). The most common genomic alterations found were *TP53* (*n* = 16, 72.7%)

Raw NGS data details are presented in additional file 2. Overall, at least one genomic alteration was found in 21 out of 22 patients. The median number of significant mutations for each patient was two (range; 0–6) (Fig. 1). No association was found between the number of mutations and any clinicopathological factor.

The most common genomic alterations found were *TP53* (*n* = 16, 72.7%), followed by *KRAS* (*n* = 6, 27.3%), *APC* (*n* = 5, 22.7%), *PIK3CA* (*n* = 4, 18.2%), *CTNNB1* (*n* = 3, 13.6%); two (9.1%) each for *KIT, BRAF, CDKN2A* and *PTEN;* and one (4.5%) each for *FBXW7,* *EGFR*, *ERBB2, ERBB4, GNSA, STK11, VHL* and *NOTCH1* (Fig. 1).

Most *TP53* mutations were identified in exons 5 to 8: exon 5 in 8 cases, exon 6 in 5 cases, exon 7 in 6 cases, and exon 8 in 5 cases. *KRAS* mutations were found in 6 cases, 5 in codon 12 and 1 in codon 61. Five tumors were well to moderately differentiated and one tumor was poorly differentiated. Five out of six patients had metastatic tumors. *BRAF* mutations were found in 2 cases and neither included the V600E mutation. *KRAS* and *BRAF* mutations were mutually exclusive. *APC* mutations were found in 5 patients. Four patients had a single *APC* mutation and one patient had two different *APC* mutations. All *APC* mutations were found to be nonsense or frameshift mutations and distributed between codons 700 and 1200, or between codons 1400 and 1600, consistent with previous data. All tumors with *APC* mutations were well differentiated or moderately differentiated. The *APC* mutation rate was significantly higher in pT4 than in pT1-3 but tended to be lower in metastatic tumors (Table 2). There was no significant association found between distant metastases and any single gene mutation.

**Table 2:** Relationship between common genetic alterations and clinicopathological factors of patients with SBA

		***TP53***		***KRAS***		***APC***		***PIK3CA***		***CTNNB1***	
	No.	No. (%)	*P*	No. (%)	*P*	No. (%)	*P*	No. (%)	*P*	No. (%)	*P*
**Site**											
Duodenum	3	3 (100)	NS	0	NS	1 (33)	NS	0	NS	0	NS
Jejunum	16	12 (75)		4 (25)		3 (19)		4 (25)		2 (13)	
Ileum	3	1 (33)		2 (67)		1 (33)		0		1 (33)	
**Histology**											
Differntiated	15	12 (75)	NS	5 (33)	NS	5 (33)	NS	3 (20)	NS	1 (7)	NS
Por	5	3 (60)		1 (20)		0		1 (20)		1 (20)	
Muc	2	1 (50)		0		0		0		1 (50)	
**mucin phenotype**											
Gastric type	2	2 (100)	NS	0	NS	0	NS	1 (50)	NS	0	NS
GI type	3	1 (33)		1 (33)		0		1 (33)		0	
Intestinal type	12	9 (75)		4 (33)		4 (33)		1 (8)		2 (17)	
Null type	5	4 (80)		1 (20)		1 (20)		1 (20)		1 (20)	
**pT**											
pT1-3	20	15 (75)	NS	5 (25)	NS	3 (15)	0.043	4 (20)	NS	3 (15)	NS
pT4	2	1 (50)		1 (50)		2 (100)		0		0	
**pN**											
pN0	10	9 (90)	NS	1 (10)	NS	3 (30)	NS	3 (30)	NS	1 (10)	NS
pNx	12	7 (58)		5 (42)		2 (17)		1 (8)		2 (17)	
**M factor**											
negative	15	13 (87)	NS	4 (27)	NS	4 (27)	NS	3 (20)	NS	2 (13)	NS
positive	7	3 (43)		2 (29)		1 (14)		1 (14)		1 (14)	
**TNM stage**											
l	1	1 (100)	NS	0	NS	0	NS	0	NS	0	NS
ll	9	8 (89)		1 (11)		3 (33)		3 (33)		1 (11)	
lll	5	4 (80)		3 (60)		1 (20)		0		1 (20)	
lV	7	3 (43)		2 (29)		1 (14)		1 (14)		1 (14)	
**MMR status**											
pMMR	16	11 (69)	NS	4 (25)	NS	2 (13)	NS	2 (13)	NS	3 (19)	NS
dMMR	6	5 (83)		2 (33)		3 (50)		2 (33)		0	

*dMMR* DNA mismatch repair-deficient, *pMMR* DNA mismatch repair-proficient

### Relationship between gene mutations and MMR status

*APC* mutations tended to be higher in dMMR tumors than in pMMR tumors. *CTNNB1* mutations were not found in dMMR tumors. There was no significant association found between MMR status and any single gene mutation (Table 2).

### Comparison of gene mutations with immunostaining of their gene products

The p53 immunostaining pattern was diffuse and strong in all *TP53* mutation cases, and sparse and weak in all *TP53* wild-types. Aberrant β-catenin immunostaining in cancer cells was observed in 9 out of 22 cases, of which 3 cases had the *CTNNB1* mutation, one case had the *APC* mutation, and the remaining 5 cases had no mutations with Wnt pathway associated genes. HER2 immunostaining was negative in all cases examined.

### Relationship between gene mutations and mucin phenotypes

*APC* and *CTNNB1* mutations were not found in G-type and GI-type tumors. *KRAS* mutations were found in all tumor types except for G-type tumors. *TP53* mutations were found in all tumor types (Table 2).

### Survival analysis

**Fig. 2:**
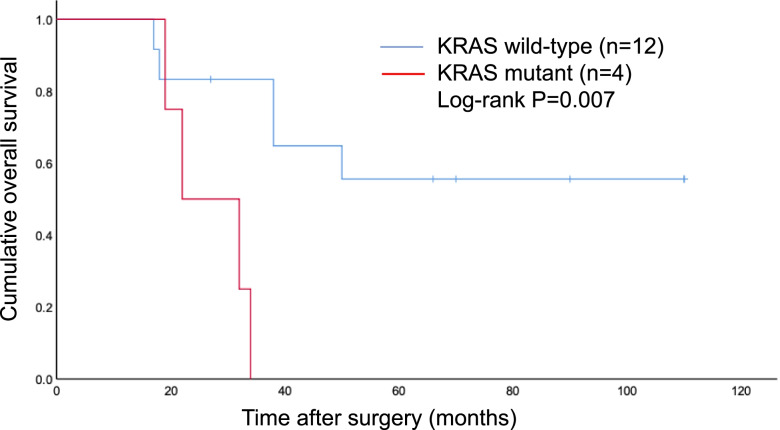
Kaplan–Meier overall survival curve for KRAS mutations in MMR proficient patients. Survival rates were significantly worse in patients with *KRAS* mutations according to log-rank tests

No single gene mutation was associated with overall survival. On the other hand, survival rates in patients with dMMR tumors were significantly better. Since the mortality rate in patients with dMMR tumors was 0, we only analyzed survival rates in the subgroups of patients with pMMR tumors. Survival rates were significantly worse in patients with *KRAS* mutations according to log-rank tests (Fig. 2).

## Discussion

In the present study, we performed a comprehensive gene mutational analysis of SBA in Japanese patients by NGS. To date, two large cohort studies regarding genomic profiling of SBA have been reported, one from the USA and the other in France [10, 12]. There has also been a relatively large cohort study on genomic profiling of SBA reported from China [15]. To our knowledge, this is the first effort to characterize genomic alteration in Japanese SBA patients using NGS.

In this study, we found 17 shared mutated genes in SBA, and identified *TP53* as the most commonly altered gene, followed by *KRAS*, *APC*, *PIK3CA*, and *CTNNB1*. Our results differ somewhat from those of previous reports, where *TP53*, *KRAS*, *APC*, and *SMAD4* were found to be the common genes altered in SBA [10–12]. We failed to detect *SMAD4* mutations in SBA patients; instead, the *CTNNB1* mutation occurred more frequently. Our results also differ from a previous report from China, in which *APC* mutations were higher and *TP53* mutations were lower than in our study [15]. We think that these differencies do not necessarily reflect racial differences.

We found that the frequency of *TP53* mutations was 72.7%, a higher ratio than reported by others [9–12, 15]. Studies have also shown that *TP53* mutation rates are lower in the duodenum than in the jejunum or ileum [11, 12]. Usually, duodenal cancers account for roughly half of all small intestinal cancers [4]. In this study, however, the ratio of duodenal cancers was significantly lower, with 3 out of 22 (14%) small intestinal cancers originating in the duodenum, which may be site-biased. There are also conflicting data regarding the association between *TP53* and prognosis, although there have been a few studies on *TP53* mutation and prognostic significance in SBA patients. Alvi et al*.* found that *TP53* mutation significantly associated with poor prognosis [9], while other researchers did not find any significant relationship between *TP53* mutation and patients’ prognosis [21]. Here too, we found no significant association between *TP53* mutation and prognosis. Moreover, Aparicio et al*.* found that *TP53* mutation frequency tended to be lower in dMMR tumors [12], whereas we found no association between *TP53* mutation and MMR status.

The *KRAS* mutation rate was relatively lower in this study compared to some reports by others [9–12, 15], though it did correspond to reports from Japan [22]. Jun et al*.* reported that a *KRAS* mutation rate of 27% in the jejunum and ileum, which is almost identical to our results [23]. Aparicio et al*.* reported that the *KRAS* mutation rate of SBA was 44% in the entire small intestine, but 32.1% in the jejunum [12]. They also reported that the *KRAS* mutation rate was significantly higher in the duodenum compared to the jejunum and ileum [12]. In this study, the jejunum accounted for a large proportion (73%) in the study, which may be site-biased. Taken together, the data suggest that there are site-specific differences in *KRAS* mutations. Most studies have identified *KRAS* mutations in codon 12, consistent with our results [23–26]. Although Jun et al*.* reported that *KRAS* mutations associated with poor prognosis in pT1-3 tumors [23], we found that *KRAS* mutations associated with poor prognosis in pMMR patients, but not in the entire cohort of SBA patients.

*BRAF* mutations in SBA have been reported to be comparable to those in CRC, but the V600E mutation has been shown to be rare in SBA [9–12]. We found no *BRAF* V600E mutations in this study, consistent with previous reports. *BRAF* mutations are more likely to be found in the right sided colon, which has been suggested to be associated with carcinogenesis originating from sessile serrated lesions (SSL) [27]. *BRAF* mutation rates are low even in the ileum, which is anatomically close to the right sided colon, an indication that carcinogenesis associated with SSL play a limited role in the small intestine.

*APC* mutations have been reported to be much lower in SBA compared to CRC [10], corresponding with our results. Furthermore, all five patients with *APC* mutations had other concurrent gene mutations (*TP53* or *KRAS*). *CTNNB1* mutations have been shown to closely associate with activation of the Wnt pathway as well as *APC* mutations [28]. The *CTNNB1* mutation rates were 13.6% in SBA in this study. These results suggest that the Wnt pathway is not as involved in SBA as it is in CRC and thus *APC* mutations on their own are not sufficient to evoke carcinogenesis of SBA. The Wnt pathway is the main carcinogenetic pathway of CRC, where adenoma present as precancerous lesions [29]. Since adenoma is found infrequently in the small intestine except for the duodenum, the adenoma-carcinoma sequence seems not to be a major pathway of carcinogenesis in SBA [30]. Adenoma is relatively common in the duodenum compared to the jejunum and the ileum [30]. Although the *APC* mutation rates in intestinal-type non-ampullary duodenal adenoma are similar to colorectal adenoma, *APC* mutations have been found to be rarer in advanced duodenal carcinoma [31]. This strongly contradicts the hypothesis that adenocarcinoma of the duodenum develops from adenoma. From this perspective, adenoma of the small intestine may not represent a precancerous lesion, in contrast to adenoma of the colon and rectum. We performed immunohistochemical analysis for β-catenin localization to determine whether there is activation of the Wnt pathway. We found dysregulation of β-catenin including loss of membranous staining and nuclear staining in all tumors exhibiting mutations of the *CTNNB1* gene. However, some β-catenin dysregulation was also found in all 5 tumors showing neither *CTNNB1* mutations nor *APC* mutations. These results suggest that the significance of the Wnt pathway may be underestimated based on gene mutation analysis alone. Alternatively, there may be differences in carcinogenesis depending on the site in the small intestine.

Although *ERBB2* mutations have been reported to be relatively high [10–12], they were not so in this study. HER2 immunostaining was also negative in all cases examined. Since studies have shown that *ERBB2* mutations are more prevalent in the duodenum, it may be that differences in *ERBB2* mutations are site-specific [8, 10, 15]. I In addition, *ERBB2* mutations have been reported to be associated with dMMR [12]. Since patients with dMMR tumors had better prognosis, it is unlikely that SBA can be a target of HER2 therapy such as trastuzumab other than for the duodenum.

Other studies have shown a relatively high mutation rate for the *SMAD4* gene [10, 12, 15], but no *SMAD4* mutations were found in this study. *SMAD4* mutations are important, particularly in pancreatic carcinogenesis, where they have been found in roughly 50% of pancreatic adenocarcinoma and 20% of invasive ampullary carcinoma [32–34]. Aparicio et al. found a 14.4% *SMAD4* mutation rate in SBA for the entire small intestine, but a rate of 7% for the jejunum [12]. In this study, we strictly excluded ampullary carcinoma. These results suggest that *SMAD4* mutations may be influenced by organ-specific differences.

To date, the relationship between mucin expression and gene mutations in SBA has yet to be investigated in detail. Kumagai et al*.* have reported that SBA cases with *KRAS* mutations were positive only for MUC2 but negative for the markers of gastric phenotypes including MUC5AC and MUC6 [25]. We detected *KRAS* mutations in 1 GI type tumors, which were positive for both MUC5AC and MUC6. These results suggest that *KRAS* mutations and gastric phenotype markers are not always mutually exclusive. On the other hand, *APC* and *CTNNB1* mutations were not found in G-type and GI-type tumors, suggesting that there is a reciprocal relationship between *APC* and *CTNNB1* mutations and gastric phenotype markers. It has been also suggested that molecular variations differ between gastric type and intestinal type SBA [31, 35]. Therefore, the mucin phenotype should also be considered when examining gene mutations of SBA.

The relationship between gene mutations and prognosis has been investigated in SBA. Alvi et al. have reported that *TP53* mutations associated with worse prognosis [9]. Jun et al*.* reported that *KRAS* mutations correlated with worse prognosis in a cohort of patients with a pT1-T3 tumors [23]. Pan H et al. have reported that *KRAS* mutations associated with recurrence-free survival [15]. However, other researchers have reported no association between genomic alteration and prognosis of SBA patients [11, 12]. We also found no correlation between gene mutations and prognosis of SBA patients. In this study, no patients with dMMR tumors died during the observational period, thus only patients with pMMR tumors underwent survival analysis. We found that *KRAS* mutations were associated with worse survival in patients with pMMR tumors.

Since SBA has been associated with Lynch syndrome, it has been suggested that microsatellite instability plays a more significant role in SBA compared to CRC or GC [36]. Studies have also shown that SBA with dMMR is characterized by less metastasis and better prognosis [12, 37]. The relationship between gene mutations and MMR status in SBA remains controversial. Aparicio et al. have reported that dMMR tumors tend to have fewer *TP53* mutations but more *ERBB2* mutations than pMMR tumors [12]. In addition, Hänninen et al. have shown that *APC* mutations were more frequent in dMMR tumors than in pMMR tumors [11]. Taken together, the relationship between gene mutations and patients’ prognosis may vary depending on the ratio of dMMR to pMMR tumors.

This study has several limitations. First, the sample size was small. In addition, there was a bias in tumor localization. Second, it included just one patient with a predisposing disease, who was diagnosed with Lynch syndrome. Third, MMR status was determined based solely on the results of immunohistochemical staining of MMR proteins.

## Conclusions

Genomic alterations in Japanese patients with SBA do not appear to differ significantly from studies conducted in Western countries. This suggests that racial differences have no substantial impact on SBA carcinogenesis. However, it does appear that HER2 plays a small role in SBA progression. It could well be that differences in patient background may account for the discrepancy among studies. Tumor localization, mucin phenotype, and MMR status should be considered as factors that influence SBA gene mutations. The characteristics of SBA are closer to those of CRC than to GC [38], which is thought to be due to the large proportion of intestinal type SBA. However, the low mutation rate of *APC* in SBA does not correspond to that in CRC. Also, in this study, there were no mutation cases involving *APC* alone. Conversely, the high *APC* mutation rate in adenoma is evidence that small intestinal adenoma is unlikely to progress to adenocarcinoma, and that the adenoma-carcinoma sequence is not the main pathway of carcinogenesis of SBA. Thus, it is necessary to consider lesions other than adenomas as precancerous lesions of SBA.

## Supplementary Information

**Additional file 1.** Relationship between MMR status and clinicopathological factors of patients with SBA.

**Additional file 2.** Raw NGS data of patients with SBA.

## Data Availability

All data generated or analyzed during this study are included in this published article and its supplementary information files.

## Abbreviations

CRC: Colorectal cancer
GC: Gastric cancer
IgG: Immunoglobulin
MMR: DNA mismatch repair
NGS: Next-generation sequencing
OS: Overall survival
SBA: Small bowel adenocarcinoma
SSL: Sessile serrated lesion
