# Supplementary material for: Genetic analysis of Japanese patients with small bowel adenocarcinoma using next-generation sequencing
Source: BMC Cancer. 2022 Jul 2;22:723. doi: 10.1186/s12885-022-09824-6 (PMC9250163; doi:10.1186/s12885-022-09824-6)
Supplement: Supplementary file 1 — Additional file 1. Relationship between MMR status and clinicopathological factors of patients with SBA. [file 12885_2022_9824_MOESM1_ESM.docx]

Additional file 1. Relationship between MMR status and clinicopathological factors of patients with SBA.

|  |  | dMMR | pMMR |  |
| --- | --- | --- | --- | --- |
|  | No. | N=6 | N=16 | P |
| Age (avr.) |  | 57.2 | 63.8 | NS |
| **Sex** |  |  |  |  |
| female | 6 | 2 (33) | 4 (67) | NS |
| male | 16 | 4 (25) | 12 (75) |  |
| **Site** |  |  |  |  |
| duodenum | 3 | 0 | 3 (100) | NS |
| Jejunum | 16 | 5 (31) | 11 (69) |  |
| Ileum | 3 | 1 (33) | 2 (67) |  |
| **Histology** |  |  |  |  |
| Differntiated | 15 | 5 (33) | 10 (67) | NS |
| Por | 5 | 1 (20) | 4 (80) |  |
| Muc | 2 | 0 | 2 (100) |  |
| **Depth** |  |  |  |  |
| pT1-3 | 20 | 6 (30) | 14 (70) | NS |
| pT4 | 2 | 0 | 2 (100) |  |
| **LN status** |  |  |  |  |
| pN0 | 10 | 4 (40) | 6 (60) | NS |
| pNx | 12 | 2 (17) | 10 (83) |  |
| **M factor** |  |  |  |  |
| negative | 15 | 5 (33) | 10 (67) | NS |
| positive | 7 | 1 (14) | 6 (86) |  |
| **TNM stage** |  |  |  |  |
| l | 1 | 0 | 1 (100) | NS |
| ll | 9 | 4 (44) | 5 (56) |  |
| lll | 5 | 1 (20) | 4 (80) |  |
| lV | 7 | 1 (14) | 6 (84) |  |
