# Supplementary material for: Genetic analysis of Japanese patients with small bowel adenocarcinoma using next-generation sequencing
Source: BMC Cancer. 2022 Jul 2;22:723. doi: 10.1186/s12885-022-09824-6 (PMC9250163; doi:10.1186/s12885-022-09824-6)
Supplement: Supplementary file 2 — Additional file 2. Raw NGS data of patients with SBA. [file 12885_2022_9824_MOESM2_ESM.docx]

Additional file 2. Raw NGS data of patients with SBA.

| Case | Gene ID | Mutation ID | Frequency | AA Mutation | CDS Mutation |
| --- | --- | --- | --- | --- | --- |
| 1 | TP53 | 10735 | 10.6 | p.R213Q | c.638G>A |
| 1 | TP53 | 11059 | 4.6 | p.C238Y | c.713G>A |
| 1 | TP53 | 44033 | 11.2 | p.T155I | c.464C>T |
| 1 | TP53 | 44391 | 8.1 | p.Y163= | c.489C>T |
| 1 | TP53 | 45479 | 6.5 | p.H168= | c.504C>T |
| 2 | TP53 | 99729 | 41 | p.R273H | c.818G>A |
| 3 | APC | 18852 | 20.8 | p.R876* | c.2626C>T |
| 3 | APC | 18734 | 12.8 | p.T1556Nfs*3 | c.4666dup |
| 3 | TP53 | 44225 | 3.5 | p.E287K | c.859G>A |
| 3 | TP53 | 10705 | 2.7 | p.R196* | c.586C>T |
| 4 | APC | 41616 | 23.8 | p.E1552* | c.4654G>T |
| 4 | PIK3CA | 774 | 24.1 | p.H1047Y | c.3139C>T |
| 4 | TP53 | 11183 | 3.5 | p.R267W | c.799C>T |
| 5 | FBXW7 | 22932 | 7.8 | p.R465C | c.1393C>T |
| 5 | APC | 18734 | 7.2 | p.T1556Nfs*3 | c.4666dup |
| 5 | KIT | 17946 | 4.6 | p.E562K | c.1684G>A |
| 5 | TP53 | 11148 | 3.3 | p.A159V | c.476C>T |
| 5 | TP53 | 44036 | 1.5 | p.S99F | c.296C>T |
| 6 | KIT | 28026 | 28.5 | p.M541L | c.1621A>C |
| 6 | PIK3CA | 163484 | 2.9 | p.E80K | c.238G>A |
| 6 | TP53 | 44603 | 3.2 | p.G279R | c.835G>A |
| 6 | TP53 | 43955 | 2 | p.V172I | c.514G>A |
| 7 | BRAF | 467 | 11.2 | p.D594G | c.1781A>G |
| 7 | KIT | 28026 | 53.5 | p.M541L | c.1621A>C |
| 8 | TP53 | 10886 | 23.6 | p.Q104* | c.310C>T |
| 8 | TP53 | 43657 | 22.2 | p.P190L | c.569C>T |
| 8 | TP53 | 99721 | 22 | p.R342* | c.1024C>T |
| 8 | KIT | 19110 | 13.3 | p.V825I | c.2473G>A |
| 8 | CDKN2A | 12501 | 12.7 | p.G122D | c.365G>A |
| 8 | CDKN2A | 12484 | 12.5 | p.D108N | c.322G>A |
| 8 | STK11 | 21359 | 11.1 | p.E199K | c.595G>A |
| 8 | CDKN2A | 13496 | 9.2 | p.V51I | c.151G>A |
| 8 | PIK3CA | 760 | 7.7 | p.E542K | c.1624G>A |
| 8 | PTEN | 5153 | 7.7 | p.Q17* | c.49C>T |
| 8 | VHL | 14408 | 7.3 | p.C162Y | c.485G>A |
| 8 | TP53 | 10771 | 5.1 | p.P250L | c.749C>T |
| 8 | TP53 | 46074 | 4.1 | p.R202C | c.604C>T |
| 8 | NOTCH1 | 12776 | 2.9 | p.Q2459* | c.7375C>T |
| 8 | EGFR | 6240 | 2.4 | p.T790M | c.2369C>T |
| 9 | KRAS | 521 | 7.8 | p.G12D | c.35G>A |
| 9 | TP53 | 10726 | 10.5 | p.E286K | c.856G> |
| 9 | TP53 | 10911 | 27.4 | p.R283C | c.847C>T |
| 9 | ERBB2 | 14065 | 3.5 | p.V842I | c.2524G>A |
| 9 | TP53 | 43606 | 3.8 | p.G245D | c.734G>A |
| 9 | TP53 | 43692 | 3.2 | p.G154S | c.460G>A |
| 9 | TP53 | 43737 | 3.6 | p.C277Y | c.830G>A |
| 9 | TP53 | 43761 | 2.5 | p.E204= | c.612G>A |
| 9 | TP53 | 43962 | 3.1 | p.S269G | c.805A>G |
| 9 | TP53 | 43987 | 2.5 | p.D208N | c.622G>A |
| 9 | TP53 | 44194 | 5.8 | p.A84V | c.251C>T |
| 9 | TP53 | 44428 | 3.5 | p.N247= | c.741C>T |
| 9 | TP53 | 44705 | 3.3 | p.H233Y | c.697C>T |
| 9 | TP53 | 46284 | 5.3 | p.G279= | c.837G>A |
| 9 | TP53 | 99668 | 3.9 | p.R196* | c.586C>T |
| 9 | TP53 | 99933 | 3.1 | p.R273C | c.817C>T |
| 10 | CTNNB1 | 5667 | 2.5 | p.S45F | c.134C>T |
| 10 | TP53 | 99933 | 3.2 | p.R273C | c.817C>T |
| 10 | TP53 | 129851 | 2 | p.H179Y | c.535C>T |
| 10 | TP53 | 129859 | 4 | p.P152L | c.455C>T |
| 11 | APC | 13125 | 16.2 | p.R1114* | c.3340C>T |
| 11 | TP53 | 44599 | 1.9 | p.R196Q | c.587G>A |
| 12 | TP53 | 43903 | 53.3 | p.V157G | c.470T>G |
| 12 | KRAS | 554 | 51.4 | p.Q61H | c.183A>C |
| 13 | KRAS | 517 | 51.4 | p.G12S | c.34G>A |
| 13 | TP53 | 43871 | 7.2 | p.R249M | c.746G>T |
| 14 | KRAS | 520 | 23.9 | p.G12V | c.35G>T |
| 14 | TP53 | 10808 | 33.4 | p.Y163C | c.488A>G |
| 15 | CTNNB1 | 1717883 |  | p.A21_A152del | c.60_455del396 |
| 15 | BRAF | 272639 | 24.6 | p.D594N | c.1780G>A |
| 16 | No hotspot |  |  |  |  |
| 17 | TP53 | 10771 | 2.7 | p.P250L | c.749C>T |
| 17 | TP53 | 43704 | 2.3 | p.C135= | c.405C>T |
| 17 | TP53 | 44300 | 6.2 | p.S183L | c.548C>T |
| 17 | TP53 | 44971 | 5.7 | p.H178= | c.534C>T |
| 17 | TP53 | 45103 | 2.7 | p.K164= | c.492G>A |
| 17 | TP53 | 45622 | 5.7 | p.G154D | c.461G>A |
| 17 | TP53 | 45627 | 4.8 | p.I162= | c.486C>T |
| 17 | TP53 | 45671 | 3 | p.R174M | c.521G>T |
| 18 | TP53 | 11059 | 32.6 | p.C238Y | c.713G>A |
| 18 | TP53 | 43700 | 41.3 | p.C238S | c.712T>A |
| 18 | TP53 | 45677 | 35.8 | p.C238* | c.714T>A |
| 18 | GNAS | 123397 | 2.7 | p.R844C | c.2530C>T |
| 19 | KRAS | 521 | 15.9 | p.G12D | c.35G>A |
| 19 | TP53 | 6932 | 29.3 | p.G245S | c.733G>A |
| 19 | TP53 | 43776 | 3.3 | p.E287= | c.861G>A |
| 20 | CDKN2A | 12473 | 17.8 | p.R58* | c.172C>T |
| 20 | PIK3CA | 775 | 21.6 | p.H1047R | c.3140A>G |
| 20 | PIK3CA | 764 | 2.3 | p.E545G | c.1634A>G |
| 21 | CTNNB1 | 5738 | 2.5 | p.A21T | c.61G>A |
| 21 | ERBB4 | 110095 | 3.1 | p.S341L | c.1022C>T |
| 21 | PTEN | 5149 | 3 | p.Q171* | c.511C>T |
| 22 | APC | 18852 | 10.1 | p.R876* | c.2626C>T |
| 22 | KRAS | 521 | 8.6 | p.G12D | c.35G>A |
